# Application of ultrasound in the closed reduction and percutaneous pinning in supracondylar humeral fractures

**DOI:** 10.1186/s13018-021-02755-1

**Published:** 2021-10-12

**Authors:** Yang Wu, Rongbin Lu, Shijie Liao, Xiaofei Ding, Wei Su, Qinjun Wei

**Affiliations:** Department of Orthopedic Trauma and Hand Surgery, The First Affiliated Hospital of Guangxi Medical University, No.6 Shuangyong Road, Nanning, Guangxi China

**Keywords:** Supracondylar humeral fractures, Ultrasound, Closed reduction, Percutaneous pinning

## Abstract

**Background:**

Ultrasound examination can be applied to the diagnosis of pediatric elbow fracture. This study aims to analyze the application value of ultrasound in the surgical treatment of supracondylar humeral fractures.

**Methods:**

64 children with supracondylar humeral fractures were treated with ultrasound-guided closed reduction and percutaneous pinning (CRPP), 31 patients were treated with CRPP under radiography guidence. The reduction effect of supracondylar humeral fractures was determined through the perioperative ultrasound images of the lateral, medial and posterior aspects of the elbow. Percutaneous pinning was performed after supracondylar humeral fractures were well reduced. A follow-up examination was performed and all the patients were evaluated according to Flynn’s criteria.

**Results:**

The mean duration of surgery was 58.3 min (42–108 min) in the ultrasound group and 41.5 min (24-63 min) in the radiography group (*P* < 0.05). The mean carrying angle was 8.2° (0°–15°) in the ultrasound group and 9.4°(3°–16°) in the radiography group; The mean Baumann’s angle was 75.5°(60°–85°) in the ultrasound group and 73.4°(62°–82°) in the radiography group; The mean lateral humerocapitellar angle was 38.4° (26°–54°) in the ultrasound group and 41.6°(29°–52°) in the radiography group; No significant differences were observed between the two groups. According to the Flynn’s criteria, 49 (76.6%) patients had excellent, 10 (15.6%) patients achieved good, 3 (4.7%) patients showed fair results and 2 (3.1%) patients achieved poor results in the ultrasound group; 22 (70.9%) patients had excellent, 6 (19.4%) patients achieved good, 2 (6.5%) patients showed fair results and 1 (3.2%) patients achieved poor results in the radiography group; No statistically significant difference was noted between the results of these two groups (*P* > 0.05). After surgery, three patients had pin tract infection. One patient had ulnar nerve neurapraxia in the radiography group. No cases with Volkmann’s contracture were reported.

**Conclusion:**

Ultrasound-guided CRPP is a safe and reliable surgical treatment of pediatric supracondylar humeral fractures.

*Trial registration* Retrospectively registered.

## Introduction

Supracondylar humeral fractures are the most-common elbow fractures in children, accounting for 60–70% of pediatric elbow fractures, which mainly affect children in 5–8 years [1–3]. The occurrence of pediatric supracondylar humeral fractures has a close relation to the season, and the incidence is high in summer. They are usually caused by hyperextension or flexion violence from falling during activities. After falling from high place, the outstretched hand touching the ground suffers a violence transmitted to the weak olecranon fossa, thus causing a fracture. Supracondylar humeral fractures are classified into the extension type (98%) and flexion type (2%) [4]. The extension type of supracondylar humeral fractures is caused by elbow hyperextension and the distal end of the fracture is displaced backward and upward; While the flexion type is caused by elbow flexion with the olecranon fossa touching the ground, and the distal end of the fracture is displaced forward and upward. According to the Gartland classification, extension supracondylar humeral fractures are categorized into four types. Type I: Fracture is nondisplaced, which is treated by cast immobilization; Type II: Fracture presents slight displacement with a posterior humeral cortical contact (IIa) or when the fracture presents a straight or rotatory displacement with contact between the two fragments (IIb); Type III: Fractures have a posteromedial or posterolateral displacement associated with a loss of integrity of the posterior cortex; Type IV: Fractures with multidirectional instability [4]. Supracondylar humerus fractures may cause severe acute morbidity and complications, such as nerve injury and vascular injury.

In 1948, Swenson first reported the treatment of supracondylar humeral fractures by closed reduction with K-wire fixation [5]. Closed reduction and percutaneous pinning(CRPP) under the guidance of the intraoperative radiographs is an effective therapeutic strategy for supracondylar humeral fractures [6]. However, intraoperative radiographs increase the exposures to both patients and operators. To reduce radiation exposures, we adopted intraoperative ultrasound to guide the CRPP. Multiple ultrasound images can identify a hinge of soft tissue at the fracture site. Stress testing and the use of ultrasound scanner are also helpful in assessing the stability of the reduction.

## Materials and methods

In the present study, a total of 95 children with supracondylar humeral fractures who were treated by CRPP in our hospital from 2017 to 2019 were retrospectively analyzed. 64 patients were treated with CRPP under ultrasound guidence, 31 patients were treated with CRPP under radiography guidence. This study was approved by the institutional review board. Informed consent was obtained from family members. Inclusion criteria: Patients below 10 years; Type II, III or IV supracondylar humeral fractures. Exclusion criteria: Undisplaced Gartland type I fractures; Pathological fractures; Open fractures; Multiple compound injuries; Follow-up of less than 1 year.

**Fig. 1:**
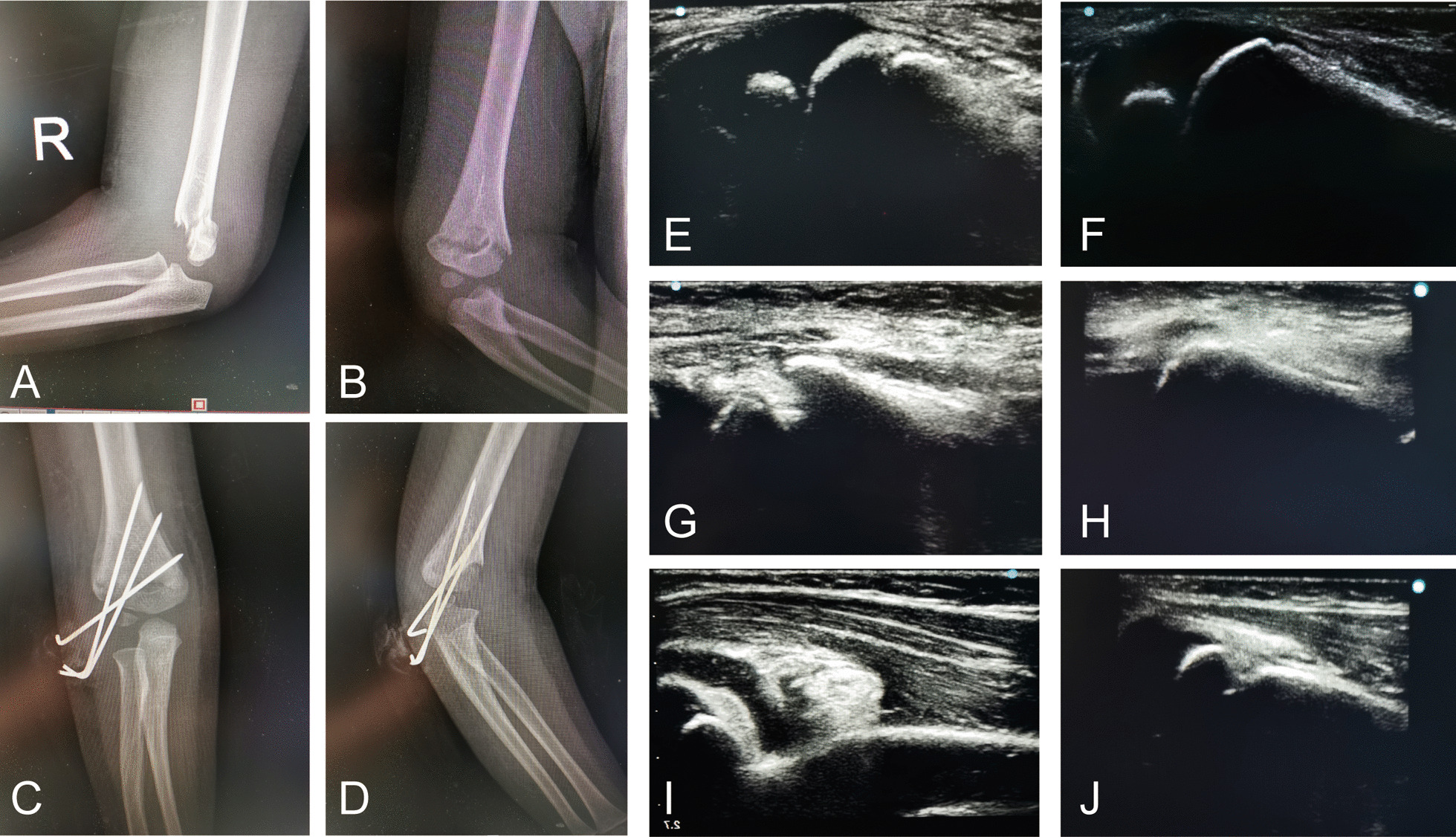
**a** Preoperative lateral radiogram of the patient with Gartland II humeral fracture; **b** Preoperative anteroposterior radiogram. **c** Postoperative anteroposterior radiogram after CRPP; **d** Postoperative lateral radiogram after CRPP. **e** Lateral, **g** medial and **i** posterior sonogram of forearm shows softtissue swelling around elbow and medial displacement of distal humerus; **f** Lateral, **h** medial and **j** posterior sonogram after reduction shows distal humerus back in its normal position

**Fig. 2:**
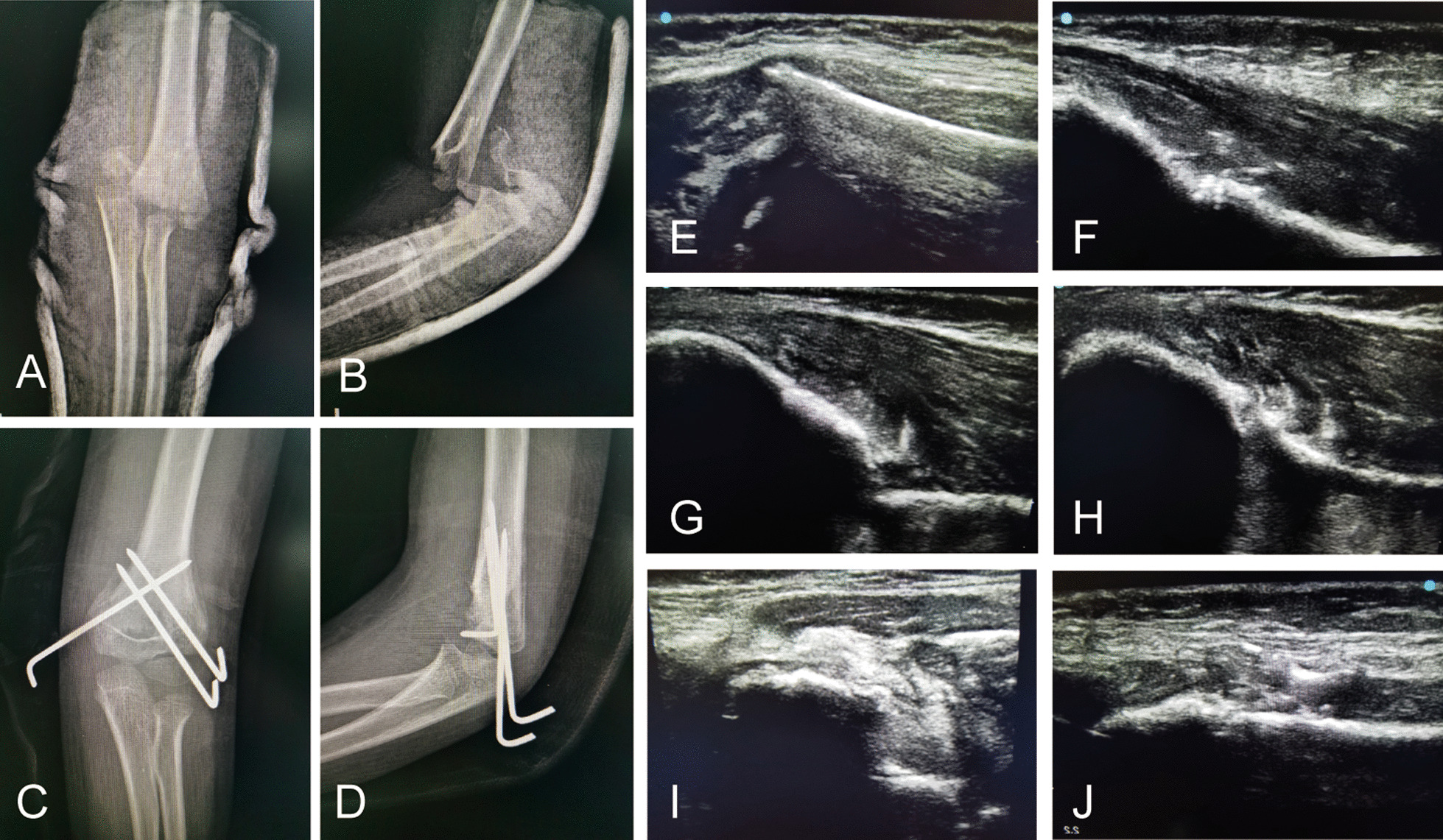
**a** Preoperative anteroposterior radiogram of the patient with Gartland III humeral fracture; **b** Preoperative lateral radiogram. **c** Postoperative anteroposterior radiogram after CRPP; **d** Postoperative lateral radiogram after CRPP. **e** Lateral, **g** medial and **i** posterior sonogram of forearm shows softtissue swelling around elbow and medial displacement of distal humerus; **f** Lateral, **h** medial and **j** posterior sonogram after reduction shows distal humerus back in its normal position

All children with supracondylar humeral fractures were surgically treated within 6–72 h by the same operator. The patient was placed in a supine position after general anesthesia, and the fractured limb was extended, sterilized and draped. Closed reduction was performed under the guidance of the Sonosite SII ultrasound. Gentle traction was applied to the forearm, and the elbow was extended gradually to reduce the angulation. Ultrasound probe was placed on the lateral and medial elbow, and the operator's thumb pushed the fracture of the distal humerus to correct the lateral displacement of the fracture. Next, the elbow was flexed while pushing the olecranon with the thumb to correct the posterior displacement of the distal humeral epiphysis. At the same time, the forearm was pronated or supinated to correct the rotation of the fragment. A complete assessment of the elbow requires longitudinal images of the joint: posterior, medial and lateral (Figs. 1 and 2). The radial and medial side ultrasonography showed the lateral displacement of the fracture; The posterior ultrasonography showed the posterior displacement of the fracture. The fracture was fixed either by the crossed or the lateral method. After the intraoperative reduction and fixation were completed, intraoperative radiographs are needed for the placement of fixation pins. The pins are bent to lie against the skin and cut leaving approximately 2–3 cm to prevent pin migration and facilitate pin removal when healing occurs.

All patients returned for clinical evaluations at 1w, 4w, 6w and 6 m, 12 m and 24 m (Fig. 1 and 2). The cast and pins were removed after fracture union in the clinic. Physical examinations were performed, including neurovascular examination, carrying angle and the motion range of elbow flexion and extension. Radiographic evaluation included the Baumann’s angle and humerus anteversion angle. Elbow joint function was assessed by Flynn’s criteria (Based on the carrying angle and the range of elbow motion) [7].

### Statistical analysis

T tests were used to compare continuous variables. Comparison between the study groups was performed using x^2^ equation. *P* < 0.05 was considered statistically significant. SPSS (Statistical Package for the Social Science; SPSS Inc, Chicago, IL) was used for data analysis.

## Results

Among the 95 recruited patients, there were 54 boys and 41 girls, with the mean age of 5.0 years (2.4–10 years). The left elbow was involved in 58 patients and the right in 37 patients. In addition, according to the Gartland classification, there were 26 cases with Gartland II and 69 with Gartland III. 95 recruited patients with supracondylar humeral fractures were followed up for 16.2–24 months (mean: 19.4 months). The mean duration of surgery of was 58.3 min (42–108 min) in the ultrasound group, the mean duration of surgery of was 41.5 min (24–63 min) in the radiography group. Ultrasound-assisted reduction took longer than fluoroscopic surgery (*P* < 0.05, Table 1).

**Table 1:** Demographic and clinical parameters of children with supracondylar humeral fractures

Parameters	Ultrasound group	Radiography group	*P* value
Age (years)	5.2 (2.5–10)	4.8 (2.4–9.5)	0.79
Sex (male/female)	35/29	19/12	0.54
Fracture side (L/R)	38/26	20/11	0.63
Duration of surgery (min)	58.3 (42–108)	41.5 (24–63)	0.02
Average length of stay (Day)	3.2 (1.5–6.4)	2.8 (1.2–5.7)	0.51

The mean carrying angle was 8.2° (0°–15°) in the ultrasound group and 9.4°(3°–16°) in the radiography group; The mean Baumann’s angle was 75.5° (60°–85°) in the ultrasound group and 73.4° (62°–82°) in the radiography group; The mean lateral humerocapitellar angle was 38.4° (26°–54°) in the ultrasound group and 41.6° (29°–52°) in the radiography group; No significant differences were observed between the two groups (Table 2). According to the Flynn’s criteria, 49 (76.6%) patients had excellent, 10 (15.6%) patients achieved good, 3 (4.7%) patients showed fair results and 2 (3.1%) patients achieved poor results in the ultrasound group; 22 (70.9%) patients had excellent, 6 (19.4%) patients achieved good, 2 (6.5%) patients showed fair results and 1 (3.2%) patients achieved poor results in the radiography group; No statistically significant difference was noted between the results of these two groups (*P* > 0.05, Table 3).

**Table 2:** Follow-up data for children with supracondylar humeral fractures

Parameter	Ultrasound group	Radiography group	*P* value
Pin tract infection (%)	3.1	3.2	0.98
Extension ranges (°)	2.1° (− 4° to 8°)	3.4° (− 5° to 9°)	0.49
Flexion ranges (°)	142.3° (122°–157°)	146.1° (125°–154°)	0.58
CA of the injured elbow (°)	8.2° (0°–15°)	9.4° (3°–16°)	0.64
BA of the injured elbow (°)	75.5° (60°–85°)	73.4° (62°–82°)	0.72
LHA of the injured elbow (°)	38.4° (26°–54°)	41.6° (29°–52°)	0.47

*BA* Baumann angle, *CA* Carrying angle, *LHA* Lateral humerocapitellar angle

**Table 3:** Outcomes according to Flynn’s criteria

Outcomes	Ultrasound group	Radiography group	*P* value
Loss of motion [n (%)]			
Excellent	53 (82.8)	24 (77.4)	0.93
Good	6 (9.4)	4 (12.9)	
Fair	3 (4.7)	2 (6.5)	
Poor	2 (3.1)	1 (3.2)	
Loss of carrying angle [n (%)]			
Excellent	56 (87.5)	26 (83.9)	0.83
Good	7 (10.9)	4 (12.9)	
Fair	1 (1.6)	1 (3.2)	
Poor	0	0	

After surgery, pin tract infection was observed in 3 patients; 2 of them from the ultrasound group and 1 from the radiography group. All cases were cured after removal of K-wires at 4 weeks postoperatively. One patient had ulnar nerve neurapraxia in the radiography group, and it was recovered in the sixth week postoperatively. In ultrasound group, one radial nerve lesion was observed in the first physical examination at admission and resolved spontaneously after 6w; One patient had no pulse of radial artery but good limb perfusion after fracture reduction, the patient was admitted for observation and had a palpable pulse return without clinical sequelae.

## Discussion

Most children with supracondylar humeral fractures initially received closed manipulation without imaging assistance in the emergency departments. Gartland type III supracondylar fractures may require CRPP. Closed manipulation without image assistance can require repeated reductions and multiple post-manipulation radiographs. This increases patient’s pain, radiation exposure and may lead to swelling at the fracture site. The use of fluoroscopy can avoid these drawbacks, but many emergency departments do not have these units available. Therefore, a real-time imaging technique is required to assist the reduction. Ultrasound is widely used in many hospitals as an inexpensive, radiation-free device that provides real-time images. Compared with fluoroscopy units, the ultrasound units are smaller and easier to set up in a crowded operating room. Most orthopedic surgeons do not have a training background in Sonography. To standardize this procedure, physicians should participate after receiving training in performing ultrasound-guided CRPP. Previous studies have reported the importance of ultrasonography in pediatric elbow joint injuries, which is able to assess the anatomical relationship between elbow joint muscles and nerves [8]. Barr and Davidson reported that ultrasound is a reliable examination for the elbow joint that clearly visualizes the distal humeral epiphysis, olecranon fossa and coronal fossa [9, 10]. Most importantly, ultrasound examination accurately reveals the epiphysis of infants and young children. Markowitz et al. analyzed the normal ultrasound images of the elbow joint from four aspects and fully reflected the relationship between the humerus, the humeral joint, and the humeroulnar joint [11]. Grechenig reported that the ultrasound could detect cortical discontinuities of 1 mm or more [12]. Kotlarsky showed that ultrasonography-guided forearm fracture reduction was an effective and useful method for the correction of displaced forearm and wrist fractures in children [13]. A study of radiation exposures in 248 children reported that the mean DAP exposure of supracondylar fractures was 22.3 mGy/cm^2^ [14]. Ultrasound-assisted reduction provides real-time images of fracture displacement, helping the surgeon to manipulate the fracture segment without radiation exposure.

In order to demonstrate accurately the various anatomical features of the joint, ultrasonography of the elbow must include multipie images in different planes. Davidson reported that posterior and lateral sonograms are the most important, and that anterior sonograms are not helpful in the evaluation of a fractured elbow [10]. In this study, closed reduction was performed under the guidance of ultrasound, and the displacement was observed from the posterior, lateral and medial aspects. Under normal circumstances, the cortical bone and epiphysis are continuous and smooth on the ultrasound image of the elbow joint. The hyperechoic area in the center is the ossification center displayed in the capitate eminence, while the peripheral hypoechoic area is the epiphysis [10, 11]. The anterior cortex of humerus is hyperechoic. An extended line along the hyperechoic shadow of the humeral cortex is called the anterior humeral line, which passes through the anterior third of the capitulum of the humerus. Once supracondylar humeral fractures occur, ultrasound examination reveals a disrupted continuity of the cortical bone. The fat pad sign can be observed in the olecranon. The posterior medial displacement of the distal end of the fracture is more common than the posterolateral displacement in the extension type of supracondylar humeral fractures, and the posterior periosteum and joint capsule are basically intact. The sagittal image of the posterior part of the medial third of the distal end of the humerus was helpful for the visualization of fractures, because the distal fracture fragment usually was displaced posteriorly. It is very important to identify the displacement direction of the fracture, which determines adjacent tissues punctured by the fracture. Ultrasound examination can accurately assess the displacement direction of the fracture, and the integrity of the periosteum and the joint capsule. To assess the ultrasonographic appearance of the posterior aspect of the distal end of the humerus and the supracondylar fossa, it is helpful to think of the distal part of the humeral shaft as a shallow spoon that contains the echogenic fat pad. Ultrasonography is non-invasive and can be repeatedly examined, which is greatly conductive to the adjuvant treatment of supracondylar humeral fractures. Gadgil reported that elevated, straight-arm traction is safe and effective in children younger than ten years [15]. Boyd reported that the successful rate of fluoroscopy-guided reduction in the treatment of supracondylar humeral fractures was 94% [16]. In this study, 22 (70.9%) patients had excellent, 6 (19.4%) patients achieved good results in the radiography group. Zhou reported that the successful rate of ultrasonography-guided closed reduction in the treatment of distal humeral transphyseal fractures was 84% [17]. In this study, 49 (76.6%) patients had excellent, 10 (15.6%) patients achieved good results in the ultrasound group. Elevated, straight-arm traction can be effectively used in an environment that can provide ordinary paediatric medical care and general orthopaedic expertise. But, some weeks of inpatient management of children can impose a burden on their parents. In this study, the average length of stay was 3.2 days in the ultrasound group. But elbow ultrasound is still considered an operator-dependent procedure and it requires an experienced operator and continuous clinical feedback in order to achieve effective reduction. In comparison with the studies of fluoroscopy-guided reduction, the overall excellent to good results in the ultrasound group is equal to the average of 90–95%. Our data suggest that ultrasound assistance can aid reduction of supracondylar humeral fractures as well as fluoroscopy.

Under the guidance of ultrasound, K-wire fixation can avoid the ulnar nerve injury and improve the safety of closed reduction. The incidence of iatrogenic ulnar nerve injury in the treatment of supracondylar humeral fractures ranges 2–3% [18]. Rasool et al. reported that through surgical exploration, ulnar nerve injury is rarely directly caused by fractures, but more often by the compression of the ulnar nerve and surrounding soft tissues caused by K-wires, which is also supported by ultrasound findings in Karakurt's study [19, 20]. How to prevent ulnar nerve injury, rather than how to treat it after nerve injury should be highlighted. Flynn recommended touching the medial epicondyle as a landmark, and Kirschner wires can be punctured in front of the medial epicondyle to avoid the ulnar nerve injury. However, Wind believed that it is not accurate enough to determine the position of the K-wires to be punctured in and the ulnar nerve only through touching the landmark of the medial epicondyle. They found that the actual mean distance from the predicted puncture site to the anatomical location of the ulnar nerve is only 2 mm [21, 22]. Even if the ulnar nerve is not directly punctured by a needle, the K-wire on the inner side that is close to the ulnar nerve may cause nerve injury. Boyd reported that 2 patients had iatrogenic ulnar nerve palsies after fluoroscopy-guided reduction. They immediately underwent open exploration. The ulnar nerve was tented around the medial pin, the medial pin was removed, all nerve palsies had completely recovered [16]. Devkota reported that seven patients got ulnar nerve injuries post-operatively. All the nerve injuries recovered within 14 weeks postoperatively except one case [23]. There was no case of iatrogenic ulnar nerve injury in this study. The ulnar nerve is located at the posteromedial aspect of the joint, which passes through the elbow region inside the cubital tunnel, and is stabilized by the Osborne ligament. Ultrasound in the axial plane is performed to assess the location of the nerve in the tunnel [24]. Owing to the peculiar internal structure, and the hypoechoic fascicles is embedded in the hyperechoic connective tissues (epineural and perineurium), the ulnar nerve is easily to be identified. The placement of K-wires under the guidance of ultrasound can educe the probability of iatrogenic ulnar nerve injury.

In clinical practice, physicians mainly determine the brachial artery damage by physical examinations on the radial artery pulsation and peripheral blood supply of the affected limb. There is no need to perform the vascular exploration after the reduction of supracondylar humeral fractures with good limb perfusion and brachial artery pulsation. Otherwise, surgical exploration is needed. However, it is controversial whether vascular exploration should be performed in children with pulseless hands but a good peripheral blood supply of limbs after reduction of supracondylar humeral fractures. Although angiography can better diagnose the vascular injury, it cannot be intraoperatively monitored. Ultrasound is a convenient and non-invasive examination that directly assesses the blood flow of the brachial artery. Reigstad reported 5 cases of children with supracondylar humeral fractures and pulseless hands, and vascular exploration or reconstruction is performed during the operation. Follow-up at 1 year postoperatively found that all patients have recovered elbow joint activity, upper extremity circulation and grip strength [25]. Weller reported 20 cases of type III supracondylar humeral fractures with perfusion but no pulsation. They are treated by CRPP, and after surgery, their pulse is unable to be palpable but can be detected by Doppler ultrasound. All patients are closely observed, and the pulse is recovered postoperatively without clinical sequelae. Only one patient has poor limb perfusion during the observation period and is required to be treated by vascular repair [26]. In this study, one patient had no pulse of radial artery but good limb perfusion after fracture reduction in the ultrasound group. Meanwhile, intraoperative ultrasound examination revealed that the blood flow in the brachial artery was non-obscured, and there was no need for vascular exploration. The patient was admitted for observation and had a palpable pulse return without clinical sequelae. With the guidance of ultrasound, blood flow in the brachial artery could be closely monitored.

There are some technical limitations of ultrasound, such as bone shadowing and diminished ability to visualize deep structures. The fixation K-wires are uneasily delineated by ultrasound. After the CRPP were completed, intraoperative radiographs are still needed for the placement of fixation pins.

The study analyzed the CRPP in pediatric supracondylar humeral fractures under the guidance of ultrasound. We judged the quality of fracture reduction at the lateral, posterior and medial aspects to reduce the potential radiations. Under the guidance of ultrasound, K-wire fixation was performed by selecting a safe puncture site and preventing ulnar nerve injury. As a non-invasive and simple vascular examination method, ultrasound examination is of great significance to assess perioperative vascular injury as early as possible. Therefore, we recommended ultrasound-guided CRPP as the preferred therapeutic strategy for pediatric supracondylar humeral fractures.

## Data Availability

The datasets generated during and/or analyzed during the current study are not publicly available, but are available from the corresponding author on reasonable request.

## Abbreviations

CT: Computed tomography
MRI: Magnetic resonance imaging
CRPP: Closed reduction and percutaneous pinning
DAP: Dose area product
